# Clinical, neurophysiological, and radiological correlates of treatment response in elderly patients with epilepsy

**DOI:** 10.1055/s-0046-1829155

**Published:** 2026-10-27

**Authors:** Ozan Dörtkol, Ayşe Deniz Elmalı, Görkem Şirin, Nerses Bebek

**Affiliations:** 1 Istanbul University, Istanbul Faculty of Medicine, Department of NeurologyIstanbulTurkey; 2 Istanbul University-Cerrahpaşa, Institute of Graduate Studies in Health, Department of BiostatisticsIstanbulTurkey

**Keywords:** Electroencephalography, Aged, Epilepsy, Late-Onset Epilepsy, Magnetic Resonance Imaging

## Abstract

**Background:**

Epilepsy in elderly patients presents unique diagnostic and therapeutic challenges, with variable clinical, neuroimaging, and electroencephalographic (EEG) features. Understanding these complexities is crucial for optimizing management strategies in this growing population.

**Objective:**

To characterize the clinical, neuroimaging, and EEG features of elderly-onset epilepsy and to evaluate their association with seizure control and treatment response.

**Methods:**

We retrospectively evaluated 37 epilepsy cases with seizure onset after 65-years-old who had been followed up at a tertiary care center between January 2019 and January 2023. Clinical characteristics, EEG and magnetic resonance imaging (MRI) findings, seizure types, treatment responses, and comorbidities were systematically analyzed. Seizure syndromes were classified according to International League Against Epilepsy (ILAE) criteria.

**Results:**

Structural focal and unknown etiology epilepsy were the most common subtypes. Seizure remission for at least 36 consecutive months was achieved in 81.1% of patients, with 64.9% responding to first-line antiseizure therapy. The MRI scan revealed epileptogenic lesions in 40.5% of patients; however, no statistically significant association was found between neuroimaging findings and treatment outcomes. Some EEG abnormalities were noted, including epileptiform discharges (24.3%) and background slowing (24.3%), but no statistically significant association was found with seizure control. Convulsive status epilepticus occurred in 10.8% of patients but was generally well-managed. Psychiatric comorbidities were encountered less frequently compared with younger populations.

**Conclusion:**

Comprehensive clinical assessment, supported by neurophysiological and radiological findings, may help optimize the diagnosis and effective management of epilepsy in elderly patients.

## INTRODUCTION

With the increase in life expectancy, the geriatric population is expanding worldwide. Among the most common neurological disorders observed in the elderly, stroke, dementia, and epilepsy rank highest, respectively. 1 Epilepsy exhibits a bimodal age distribution, with a second peak occurring after the age of 60 years. However, there are relatively few studies specifically investigating epilepsy cases with seizure onset after 65-years-old. 2 3

Epilepsy in the elderly differs from that in younger individuals not only in terms of etiological, clinical, and laboratory features but also in respect to diagnostic challenges and therapeutic considerations.

Previous studies have reported that cerebrovascular diseases are the leading cause of epilepsy in the elderly. Other etiologies like traumatic brain injury, neurodegenerative disorders (i.e. Alzheimer's disease, other types of dementias), brain tumors, and central nervous system infections (i.e., encephalitis) are encountered less commonly, yet they are clinically relevant. 4 5 6 7 Diagnosing epilepsy in this population has its own specific challenges as memory problems and sensory issues may put a strain on acquiring a reliable history. The cumulative MRI findings due to increasing age and comorbidities may be misleading. Electroencephalography (EEG) itself may reveal more relevant or irrelevant findings, contributing to the diagnostic complexity.

Furthermore, treatment is not as straightforward as it is in the younger patients. The frequent coexistence of systemic diseases, the widespread use of polypharmacy and altered physiology due to aging complicate the management of epilepsy in the geriatric population, increasing the risk of adverse effects and necessitating further individualized treatment strategies. 8 9 These complexities highlight the importance of understanding which specific features of this rather fragile population must be addressed.

## Objectives

This study aims to conduct a comprehensive evaluation of epilepsy cases with seizure onset after 65-years-old by investigating epilepsy syndromes, seizure types, disease etiologies, electrophysiological and radiological findings, accompanying comorbidities, treatment modalities and efficacy, side effect profiles, and disease prognosis.

## METHODS

The present study retrospectively evaluated patients with seizure onset at or after 65-years-old, who were followed at the Epilepsy Clinic of the Department of Clinical Neurophysiology at Istanbul University's Faculty of Medicine, and who had at least two outpatient clinic visits between January 1 ^st^ , 2019, and January 1 ^st^ , 2023. Patients were identified from the epilepsy outpatient clinic database. All follow-up assessments were conducted by the same team in a face-to-face manner. Those with history of epilepsy before the age of 65 years were excluded.

The inclusion criteria were patients who met the diagnostic criteria for epilepsy. Patients with acute symptomatic seizures were excluded. Epilepsy diagnosis was made by an experienced epileptologist based on detailed medical history, neurological examination, electrophysiological assessments, and radiological findings. Seizure types and epilepsy syndromes were classified according to the methodology established by the International League Against Epilepsy (ILAE). 10

Demographic data, follow-up duration, age at epilepsy onset, epilepsy etiology, seizure types, comorbidities, and family history were recorded.

All patients included in the study underwent cranial magnetic resonance imaging (MRI) according to the epilepsy protocol, which included coronal and axial T1- and T2-weighted sequences, axial fluid-attenuated inversion recovery (FLAIR), diffusion-weighted imaging (DWI) with apparent diffusion coefficient (ADC) maps, and sagittal T1- and T2-weighted sequences.

The MRI findings were evaluated by experienced neuroradiologists and epileptologists, who categorized them into three groups: normal, epileptogenic lesions, and nonspecific lesions. Cases with cortical involvement, such as ischemic, space-occupying, gliotic, hemorrhagic, or atrophic changes, were classified as epileptogenic. Cases without cortical involvement, such as nonspecific white matter changes, cerebellar lesions, or osseous lesions, were classified as nonspecific.

All patients underwent at least one EEG examination lasting at least 30 minutes, including activation procedures such as hyperventilation and photic stimulation. The findings were evaluated by two experienced clinical neurophysiologists. In cases of discrepant interpretations, a joint reevaluation was performed and consensus was reached.

The EEG findings were classified into four groups: those with epileptiform activity (presence of spikes or sharp waves), those with background slowing (focal or diffuse), nonspecific abnormalities, and normal findings. Seizure types and frequency, history of status epilepticus, as well as etiological factors like history of stroke, dementia, brain tumors, autoimmune encephalitis, head trauma, hypoxic events, or a prior diagnosis of epilepsy in childhood were evaluated.

Additionally, patients' known systemic diseases, medications used for these conditions, psychiatric diagnoses, and family history were reviewed. For antiseizure medication (ASM) regimen, the maximum daily dosages of the first three prescribed drugs, their efficacy, side effect profiles, and reasons for drug discontinuation (if applicable) were analyzed. Follow-up outcomes of patients who discontinued drug therapy were also assessed.

Remission was defined as seizure freedom for at least 36 consecutive months under ASM. Seizure-free duration was calculated across all patients, regardless of remission status. Prior to statistical analysis, patients' clinical status was updated, and seizure outcomes assessed, according to their most recent follow-up visit to minimize the risk of unreported seizure recurrence due to incomplete data.

No interventional procedures were performed during the study. Patient confidentiality was strictly maintained throughout the study and after its conclusion. Participation in the study did not impose any financial burden on the participants. The study protocol was reviewed and approved by the Clinical Research Ethics Committee from Istanbul University's Faculty of Medicine (Approval No: 21, November 25, 2022).

As this was an exploratory study, descriptive statistics (percentages, measures of central tendency etc.) were calculated for different variables (e.g., age, sex, seizure types, medications). Prior to inferential statistical analyses, the distribution of continuous variables was assessed using the Shapiro-Wilk test. Variables showing normal distribution were expressed as mean ± standard deviation (SD). Descriptive statistics were calculated for categorical variables as frequencies and percentages. Chi-squared tests were used for group comparisons. When expected cell counts were less than 5, Fisher's exact test was applied.

## RESULTS

### Demographic and etiological findings

A total of 37 patients, aged over 65 years, who met the inclusion criteria were enrolled in the study, comprising 26 males and 11 females. The mean age at epilepsy onset was 70.9 ± 4.8 years, and the mean age at study enrollment was 71.1 ± 4.9 years ( Table 1 ).

**Table 1:** Demographic and clinical characteristics of the patients ( *n* = 37)

Characteristics	Patients (n, %)
**Sex (Male/Female)**	26/11 (70.3/29.7%)
**Stroke**	8 (21.6%)
Right MCA	3 (8.1%)
Left MCA	1 (2.7%)
Right PCA	2 (5.4%)
Left ACA	1 (2.7%)
TIA	1 (2.7%)
Mean latency from stroke to first seizure, months (mean ± SD)	19.5 ± 19
**Dementia**	8 (21.6%)
AD	4 (10.8%)
Normal pressure hydrocephalus	2 (5.4%)
Vascular dementia	2 (5.4%)
**Autoimmune limbic encephalitis**	1 (2.7%)
**Brain tumor**	6 (16.2%)
**Comorbidities**	
Hypertension	22 (59.5%)
Coronary artery disease	11 (29.7%)
Diabetes mellitus	8 (21.7%)
Parkinson's disease	4 (10.8%)
Arrhythmia / Atrial fibrillation	4 (10.8%)
Hypothyroidism	2 (5.4%)
Others (e.g., Charcot-Marie-Tooth disease, malignancy)	6 (16.2%)
**Psychiatric disorders**	6 (16.2%)
Depression	4 (10.8%)
Anxiety	1 (2.7%)
Psychosis	1 (2.7%)

Abbreviations: ACA, anterior cerebral artery; AD, Alzheimer's disease; MCA, middle cerebral artery; PCA, posterior cerebral artery; SD, standard deviation; TIA, transient ischemic attack.

The etiological distribution of the cohort is presented in Figure 1 . Epilepsy etiologies in the present study included 8 patients (21.6%) diagnosed with dementia, of which 4 had a prior diagnosis before the seizure onset, 3 were diagnosed with Alzheimer's disease (AD), and 1 with vascular dementia (VaD). Among the patients who developed cognitive symptoms after the seizure onset, 2 were diagnosed with normal pressure hydrocephalus (NPH), 1 with AD, and 1 with VaD. Seizure remission was achieved with monotherapy in 3 patients (37.5%) and dual therapy in 4 patients (50%). The remaining patient continued to experience temporal lobe seizures; however, their bilateral tonic-clonic seizures stopped with dual therapy.

**Figure 1:**
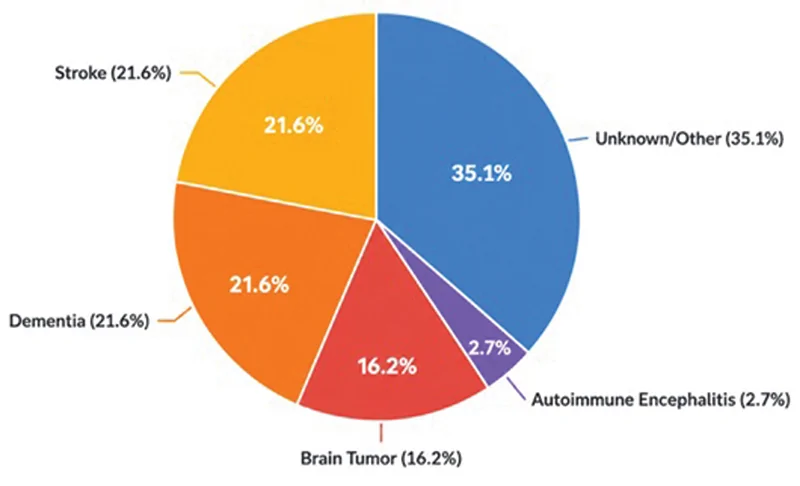
Etiological distribution of epilepsy in the study cohort.

Furthermore, another 8 patients (21.6%) had a history of ischemic stroke or cerebrovascular disease. Based on stroke localization, 4 had middle cerebral artery (MCA) involvement (50%), 2 had posterior cerebral artery (PCA) involvement (25%), and 1 had anterior cerebral artery (ACA) involvement (12.5%). All patients exhibited focal-onset seizures. Regarding outcomes, 5 patients (62.5%) achieved seizure freedom with monotherapy and 3 (37.5%) with dual therapy. The mean latency between stroke occurrence and seizure onset was 19.5 ± 19 months. One patient with a history of transient ischemic attack (TIA) experienced seizures attributed to AD pathology.

The etiology of epilepsy in 6 patients (16.2%) was brain tumors. Seizures in 2 patients with meningiomas were well controlled with appropriate ASM. However, 2 patients with diffuse glial tumors and 1 with large frontoparietal meningiomas exhibited drug-resistant epilepsy. Furthermore, one patient succumbed to malignancy. Another presented with altered consciousness and was diagnosed with nonconvulsive status epilepticus secondary to meningitis; seizures were controlled following antibiotic therapy and ASM treatment. In our cohort, patients with brain tumors appeared to exhibit a higher frequency of drug-resistant seizures; however, this observation should be interpreted cautiously given the small sample size.

Finally, one autoimmune limbic encephalitis patient with faciobrachial dystonic seizures and positive anti-LGI1 antibodies exhibited persistent seizures despite appropriate ASM therapy. Following rituximab treatment, seizure frequency decreased but temporal lobe seizures persisted approximately every 3 days. Another patient had a history of meningitis. No direct neurological or systemic cause for epilepsy could be identified in 13 patients (35.1%).

Regarding comorbidities, hypertension was present in 22 patients (59.5%), coronary artery disease in 11 (29.7%), diabetes mellitus in 8 (21.7%), Parkinson's disease in 4 (10.8%), arrhythmia in 4 (10.8%), hypothyroidism in 2 (5.4%), and other comorbidities in 6 patients ( Table 1 ).

In terms of psychiatric comorbidities, such symptoms were less frequent compared with younger patients. There were six patients (16.2%) with psychiatric comorbidities: four had depression (10.8%), one had an anxiety disorder (2.7%), and one patient with advanced Alzheimer's disease exhibited psychotic symptoms. Psychiatric symptoms were successfully managed with low-dose psychotropic drugs. No psychogenic nonepileptic seizures (PNES) were observed.

Among patients with psychiatric comorbidities, five (83.3%) were on levetiracetam, and one (16.7%) was on lamotrigine. The overall rate of levetiracetam use in the study cohort was 70.2%, though it was not statistically significant ( *p* > 0.05).

### Neuroimaging findings and electrophysiological data

Overall, epileptogenic lesions potentially responsible for seizures were identified in 15 patients (40.5%), as shown in Table 2 . Seizure remission rates according to MRI and EEG findings are illustrated in Figure 2 . Among patients with epileptogenic MRI findings, 12 (80%) achieved seizure control with appropriate ASM. Similarly, among patients with normal MRI findings, 18 of 22 patients (81.8%) achieved seizure control. There was no statistically significant difference in seizure control rates between the two groups ( *p* > 0.05). Epileptiform discharges were observed in 9 patients (24.3%), as presented in Table 2 . Organizational abnormalities were detected in 9 patients (24.3%), and nonspecific findings in 8 (21.6%). Seizure remission was achieved in 8 of the 9 patients (88.9%) with epileptiform discharges, and in 9 of the patients (75%) with normal EEG findings. There was no statistically significant difference in seizure remission rates between these groups ( *p* > 0.05).

**Table 2:** Neuroimaging and EEG findings ( *n* = 37)

Findings	Patients (n, %)
MRI	
Epileptogenic lesion	15 (40.5%)
Nonspecific	19 (51.4%)
Normal	3 (8.1%)
EEG	
Epileptiform features	9 (24.3%)
Background slowing	9 (24.3%)
Nonspecific	7 (18.9%)
Normal	12 (32.5%)
Background activity	
Good	19 (51.4%)
Adequate	9 (24.3%)
Impaired	9 (24.3%)

Abbreviations: EEG, electroencephalogram; MRI, magnetic resonance imaging.

Note: Background activity quality was assessed on baseline EEG.

**Figure 2:**
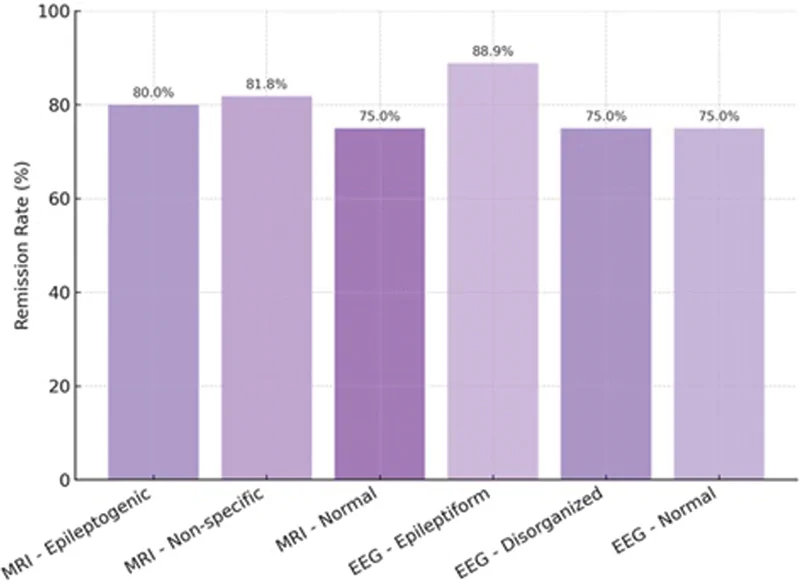
Seizure remission rates by magnetic resonance imaging and electroencephalogram findings.

Baseline EEG abnormalities were observed in 9 patients (24.3%). Among these, 5 had a history of stroke, and 4 were diagnosed with dementia. Status epilepticus was observed in 6 patients (16.2%) during follow-up, including 2 (5.4%) who developed nonconvulsive status epilepticus.

### Clinical characteristics and treatment responses

When classified according to epilepsy syndromes, 17 patients (45.9%) were diagnosed with structural focal epilepsy, 18 patients (48.7%) with unknown etiology focal epilepsy, and 2 (5.4%) with epilepsy presenting as nonconvulsive status epilepticus (NCSE). In 2 patients (5.4%), epilepsy was diagnosed solely based on nonconvulsive status epilepticus presentations, without evidence of other seizure types ( Table 3 ).

**Table 3:** Seizure types and treatment outcomes ( *n* = 37)

Clinical features	Patients (n, %)
**Epilepsy syndrome**	
Structural focal	17 (45.9%)
Unknown focal	18 (48.7%)
NCSE	2 (5.4%)
**Seizure type**	
Focal (without secondary generalization)	20 (54.1%)
Focal-to-bilateral tonic–clonic	26 (70.3%)
Status epilepticus history	6 (16.2%)
NCSE history	2 (5.4%)
Remission achieved	30 (81.1%)
**Seizure-free duration (months)**	33.5 ± 35.9
**Current treatment**	
Seizure-free with monotherapy	24 (64.9%)
Seizure-free with dual therapy	6 (16.2%)
Not seizure-free with dual therapy	6 (16.2%)
**Deceased**	1 (2.7%)

Abbreviation: NCSE, nonconvulsive status epilepticus.

Note: Remission was defined as seizure freedom for at least three consecutive years under antiseizure medication.

Among patients with unknown etiology focal epilepsy, 15 out of 18 (83.3%) achieved seizure remission, whereas 13 out of 17 (76.5%) with structural focal epilepsy achieved remission. There was no statistically significant difference between both groups ( *p* > 0.05).

Focal seizures were observed in 35 patients (94.6%), while generalized seizures (absence and myoclonic types) were reported in 2 patients (5.4%). Among patients with focal seizures, 20 (54.1%) had no secondary generalization, and 26 (70.3%) experienced focal-to-bilateral tonic–clonic seizures. No reflex seizures were observed. Despite the high frequency of focal seizures, aura was reported in only 3 patients (8.1%).

Within the focal seizure group ( *n* = 35), 14 patients (40%) exhibited frontal lobe seizures, and 18 (51.4%) had temporal lobe seizures. In three patients, seizure localization could not be determined. Seizure control was achieved in 12 of the 14 patients (85.7%) with frontal lobe seizures and in 13 of the 18 patients (72.2%) with temporal lobe seizures. No statistically significant difference in seizure control was observed between these groups ( *p* > 0.05).

In 34 patients (91.9%), follow-up lasted for at least 36 months, and all patients had follow-up durations of at least 24 months. Overall, seizure remission for at least 3 consecutive years was achieved in 30 patients (81.1%). There was one patient death (2.7%) during follow-up due to myocardial infarction. At the last follow-up, 24 patients (64.9%) were seizure-free on monotherapy. The mean seizure-free duration across the cohort was 33.5 ± 35.9 months.

Regarding the efficacy and side effects of ASMs, despite the high efficacy rates, adverse effects were more frequent and severe than expected. The most commonly prescribed initial ASM was levetiracetam (70.3%), followed by carbamazepine, lamotrigine, and phenytoin ( Table 4 ). Adverse effects developed in 6 patients (16.2%) on initial therapy. Also, seizure remission was maintained with monotherapy in 24 patients (64.9%).

**Table 4:** Antiseizure medications and adverse effects across treatment lines

Medication and side effects	Patients (n, %)	Adverse effects
**First ASD (n = 37)**		
Levetiracetam	26 (70.3%)	Irritability (3); somnolence (1)
Carbamazepine	4 (10.8%)	Tremor (1)
Lamotrigine	3 (8.1%)	
Phenytoin	2 (5.4%)	
Oxcarbazepine	1 (2.7%)	
Valproate	1 (2.7%)	Tremor (1)
**Response to first drug**	29 (78.4%)	
**Second ASD (n = 17)**		
Carbamazepine	6 (35.3%)	Dizziness (2)
Lacosamide	5 (29.4%)	
Levetiracetam	3 (17.6%)	
Oxcarbazepine	1 (5.9%)	Hyponatremia (1)
Valproate	1 (5.9%)	
Zonisamide	1 (5.9%)	
**Response to second drug**	12 (70.6%)	
**Third ASD (n = 5)**		
Lamotrigine	2 (40%)	
Lacosamide	1 (20%)	
Topiramate	1 (20%)	
Levetiracetam	1 (20%)	
**Response to third drug**	5 (100%)	

Abbreviation: ASD, antiseizure drug.

Note: Response refers to initial seizure control; sustained remission at last follow-up is reported in Table 3 . Side-effect counts in parentheses indicate the number of patients who experienced the event while receiving the specified line of therapy.

Carbamazepine was the most commonly used second-line ASM (35.3%), followed by lacosamide and levetiracetam. Among patients who received carbamazepine, two discontinued the drug due to dizziness. One patient treated with oxcarbazepine experienced hyponatremia.

With the addition of a second ASM, the remission rate increased to 86.5%. Among patients requiring a third ASM, lamotrigine was the most commonly chosen (40%). No patients required discontinuation of therapy due to drug–drug interactions.

In total, 59 ASM regimens were prescribed (45 new- and 14 old-generation ASMs).

Adverse effects leading to drug discontinuation occurred in 4 patients (28.6%) receiving old-generation ASMs and in 5 patients (11.1%) receiving new-generation ASMs. The difference between groups was not statistically significant ( *p* > 0.05).

Attempts to discontinue ASM therapy were made in four patients; however, all experienced seizure recurrence and required reinitiation of therapy. Among them, one patient with a diagnosis of meningioma had therapy discontinued at another neurosurgical center, while the remaining three patients had normal neuroimaging findings.

Status epilepticus was observed in six patients, including four with convulsive and two with nonconvulsive forms. Among patients who developed convulsive status epilepticus, three had no seizure recurrence following treatment. However, one patient with a brain tumor experienced three recurrent seizures and died due to malignancy. No seizure recurrence was observed in the two patients with nonconvulsive status epilepticus.

## DISCUSSION

In this study, etiologies differed from those typically reported, with structural and degenerative cases predominating. Atypical seizure presentations and nonspecific EEG or imaging findings highlighted the diagnostic challenges. Favorable seizure control was observed in the majority of cases, most often with monotherapy, despite frequent comorbidities and polypharmacy risks.

In our cohort, cerebrovascular disease and dementia were equally frequent, each accounting for 21.6% of cases, followed by brain tumors (16.2%) and autoimmune encephalitis (2.7%), while no clear etiology could be identified in 35.1% of patients. Previous studies in elderly populations have consistently reported cerebrovascular disease as the leading cause of epilepsy, with rates ranging between 34.1 and 39.3%, 4 and dementia as the second most frequent etiology. 4 Furthermore, stroke has been shown to increase the risk of epilepsy in elderly individuals by 8.4 to 10.6 times. 11

In contrast, in younger populations, trauma, genetic syndromes, and cortical malformations are more commonly reported as causes of epilepsy, while vascular and degenerative etiologies are relatively rare. 5 6 This discrepancy underscores the distinct etiological profile of late-onset epilepsy compared with early-onset. The relatively higher frequency of dementia in our series compared with earlier reports may be explained by referral bias, considering our center functions as a tertiary referral clinic that receives a disproportionate number of patients with cognitive disorders. However, recognition of neurodegeneration as a common and widespread etiology for epilepsy is rather recent, so these rates may reflect the true proportion of epilepsy cases due to neurodegeneration in this population. Similarly, the proportion of cases without a clear etiology (35.1%) was slightly higher than previously reported (25–40%) 5 6 which may also reflect a skewed tertiary center cohort with more complex cases.

In our cohort, focal seizures were observed in 54.1% of patients, while focal-to-bilateral tonic–clonic seizures were more frequent, occurring in 70.3% of patients. Aura was reported in only 8.1% of cases, which is lower than in younger populations. Previous studies in elderly patients have similarly shown that most seizures originate focally, with focal-to-bilateral tonic–clonic seizures accounting for approximately 65 to 75% of cases. 12 13 In younger patients, by contrast, focal seizures often remain localized, and auras are more frequently reported, reflecting better preserved seizure awareness and semiological variability. 14 The lower frequency of auras in the elderly may be related to impaired awareness, cognitive comorbidities, or rapid secondary generalization. This highlights a key difference in semiology between late- and early-onset epilepsy, with elderly patients more often presenting with generalized manifestations despite focal origins.

In our cohort, seizure remission for at least 36 consecutive months was achieved in 81.1% of patients, with 64.9% remaining seizure-free on monotherapy and 16.2% on dual therapy. Due to the increased prevalence of comorbidities and polypharmacy in the elderly, 15 drug interactions and side effects must be carefully considered. In our cohort, levetiracetam was the most frequently prescribed first-line ASM (70.3%), reflecting its low interaction potential and favorable tolerability profile. Consistent with previous studies, 14 seizure control was achieved with relatively low ASM doses, and remission was reached in 81.1% of patients. Only 16.2% of cases required 2 or more ASMs to achieve seizure control.

Furthermore, only 16.2% developed status epilepticus, including 5.4% with nonconvulsive status epilepticus, and most cases had favorable outcomes. These results are consistent with previous studies in elderly populations, which report seizure control rates of approximately 80 to 86% and emphasize the increased incidence and mortality risk of status epilepticus in the elderly. 14 16 17 18 By contrast, younger patients with new-onset epilepsy typically show lower overall remission rates and higher rates of pharmacoresistance, reflecting differences in etiology and treatment response profiles. The relatively good outcomes in our elderly cohort may be explained by the predominance of focal epilepsies secondary to vascular and degenerative processes, which appear to respond better to ASMs compared with genetic generalized epilepsies in younger patients.

Side effect rates were higher in older ASMs, leading to discontinuation in 28.6% of patients, compared with 11.1% in newer ASMs, although the difference was not statistically significant. Older age is a known risk factor for osteoporosis. 19 Moreover, older-generation ASMs have been shown to further increase fracture risk by impairing bone mineralization through cytochrome P450 enzyme induction. 20 21 These mechanisms highlight the particular vulnerability of elderly patients to ASM-related skeletal complications and reinforce the need for careful drug selection and bone health monitoring.

These findings are in line with prior reports that newer-generation ASMs, such as levetiracetam and lamotrigine, are better tolerated in the elderly due to lower interaction potential and improved side effect profiles. 22 23 A limitation of our study is the relatively small sample size, which may limit the detection of less frequent adverse effects or rare presentations of status epilepticus.

Epileptogenic lesions were identified on MRI in 40.5% of our patients, most often related to vascular or degenerative pathology, while 51.4% had only nonspecific findings and 8.1% had normal scans. On EEG, epileptiform discharges were observed in 24.3% of patients, disorganized slowing in 24.3%, and nonspecific abnormalities in 21.6%, with 32.5% showing entirely normal findings. In our cohort, no statistically significant association was observed between MRI or EEG findings and treatment outcomes.

Therefore, these findings should be interpreted cautiously and primarily as exploratory observations. Previous studies in elderly patients have similarly reported a relatively low yield of epileptiform discharges, with rates around 25 to 30%, 24 and frequent nonspecific or normal EEG results. 12 13 In younger patients, by contrast, epileptiform activity is detected more often and neuroimaging findings, particularly cortical malformations or hippocampal sclerosis, are more predictive of treatment response. These differences highlight the diagnostic challenges of late-onset epilepsy, where age-related vascular and degenerative changes may obscure or mimic epileptogenic abnormalities. The absence of a statistically significant association in our study may reflect these mechanisms, as well as limitations in conventional diagnostic tools.

The use of standardized imaging protocols and EEG reviews by two experienced neurophysiologists helped minimize misclassification. In contrast to younger patients, elderly individuals may require more extensive investigations, including autoimmune antibody panels (e.g., LGI1, CASPR2) for accurate etiological assessment. 25 Additionally, caregiver education and video recordings of events can provide valuable support for differential diagnosis in this age group. 26 Overall, individualized management strategies that account for age-specific factors may help optimize outcomes in elderly patients with epilepsy.

In our cohort, 16.2% of patients had psychiatric comorbidities, most commonly depression and anxiety, consistent with prior studies highlighting the high burden of psychiatric disorders among elderly individuals with epilepsy. Functional neurological disorders, including PNES, were not observed in our series; however, they represent an important differential diagnosis in this age group. Although no PNES cases were observed in our cohort, functional neurological disorders remain an important differential diagnosis in elderly patients with new-onset seizures. 27 28 Our findings emphasize the need for comprehensive neuropsychiatric assessment in elderly patients with new-onset seizures, as psychiatric comorbidities and functional disorders may significantly influence both diagnosis and management.

This study has several limitations. First, the sample size was relatively small, which may limit the generalizability of our findings and reduce statistical power for detecting less frequent associations. Accordingly, the absence of a statistically significant association between EEG/MRI findings and remission outcomes should be interpreted with caution, and the possibility of a type II error due to limited statistical power cannot be excluded.

Second, advanced diagnostic investigations, such as genetic investigations or autoimmune antibody panels, were not systematically performed, potentially leading to underestimation of certain etiologies. Third, seizure semiology relied partly on caregiver reports rather than video EEG monitorizations, which may have contributed to underreporting of subjective symptoms, such as aura.

Although clinical status was updated prior to analysis to reduce the risk of underreporting seizure recurrence, variability in follow-up duration should be considered when interpreting remission outcomes. Given the small sample size and subgroup distribution, the findings should be interpreted as descriptive and exploratory. Finally, our single-center design may introduce referral bias, particularly with respect to the relatively high proportion of patients with cognitive disorders. Nevertheless, the use of standardized ILAE diagnostic criteria and multidisciplinary evaluation strengthen the validity of our results.

In conclusion, in this cohort of elderly patients with epilepsy, favorable seizure control was achieved in the majority of cases, most often with monotherapy, despite frequent comorbidities and polypharmacy risks. Structural and neurodegenerative etiologies predominated, while atypical seizure presentations and nonspecific EEG or imaging findings highlighted the diagnostic challenges in this population. Overall, optimized management requires early recognition and a comprehensive, multidisciplinary approach tailored to the unique needs of elderly patients.

## References

[1] Dumurgier J Tzourio C Epidemiology of neurological diseases in older adults Rev Neurol (Paris) 2020 176 09 642 648 10.1016/j.neurol.2020.01.356 32145981

[2] Hesdorffer D C Logroscino G Benn E K Katri N Cascino G Hauser W A Estimating risk for developing epilepsy: a population-based study in Rochester, Minnesota Neurology 2011 76 01 23 27 10.1212/WNL.0b013e318204a36a PMC3032191 21205691

[3] Beghi E Giussani G Aging and the Epidemiology of Epilepsy Neuroepidemiology 2018 51(3–4):216 223 10.1159/000493484 30253417

[4] Cloyd J Hauser W Towne A Epidemiological and medical aspects of epilepsy in the elderly Epilepsy Res 2006 68 01 S39 S48 10.1016/j.eplepsyres.2005.07.016 16384689

[5] Ramsay R E Rowan A J Pryor F M Special considerations in treating the elderly patient with epilepsy Neurology 2004 62(5, suppl 2)S24 S29 10.1212/wnl.62.5_suppl_2.s24 15007161

[6] Werhahn K J Epilepsy in the elderly Dtsch Arztebl Int 2009 106 09 135 142 10.3238/arztebl.2009.0135 PMC2696249 19568380

[7] Scheffer I E Berkovic S Capovilla G ILAE classification of the epilepsies: Position paper of the ILAE Commission for Classification and Terminology Epilepsia 2017 58 04 512 521 10.1111/epi.13709 PMC5386840 28276062

[8] Fillenbaum G G Pieper C F Cohen H J Cornoni-Huntley J C Guralnik J M Comorbidity of five chronic health conditions in elderly community residents: determinants and impact on mortality J Gerontol A Biol Sci Med Sci 2000 55 02 M84 M89 10.1093/gerona/55.2.m84 10737690

[9] Charlesworth C J Smit E Lee D S Alramadhan F Odden M C Polypharmacy Among Adults Aged 65 Years and Older in the United States: 1988–2010 J Gerontol A Biol Sci Med Sci 2015 70 08 989 995 10.1093/gerona/glv013 PMC4573668 25733718

[10] Fisher R S Cross J H French J A Operational classification of seizure types by the International League Against Epilepsy: Position Paper of the ILAE Commission for Classification and Terminology Epilepsia 2017 58 04 522 530 10.1111/epi.13670 28276060

[11] Galovic M Ferreira-Atuesta C Abraira L Seizures and Epilepsy After Stroke: Epidemiology, Biomarkers and Management Drugs Aging 2021 38 04 285 299 10.1007/s40266-021-00837-7 PMC8007525 33619704

[12] Acharya J N Acharya V J Epilepsy in the elderly: Special considerations and challenges Ann Indian Acad Neurol 2014 17 01 S18 S26 10.4103/0972-2327.128645 PMC4001216 24791083

[13] Suástegui Román R A Lee Á Hernández Vanegas L E New-onset Geriatric Epilepsy in a Latin American Country: A Multi-centric Study from Mexico Rev Invest Clin 2023 75 04 203 211 10.24875/RIC.23000057 37607027

[14] Silveira D C Jehi L Chapin J Seizure semiology and aging Epilepsy Behav 2011 20 02 375 377 10.1016/j.yebeh.2010.12.033 21273137

[15] Salive M E Multimorbidity in older adults Epidemiol Rev 2013 35 75 83 10.1093/epirev/mxs009 23372025

[16] Cockerell O C Johnson A L Sander J W Shorvon S D Prognosis of epilepsy: a review and further analysis of the first nine years of the British National General Practice Study of Epilepsy, a prospective population-based study Epilepsia 1997 38 01 31 46 10.1111/j.1528-1157.1997.tb01075.x 9024182

[17] DeLorenzo R J Towne A R Pellock J M Ko D Status epilepticus in children, adults, and the elderly Epilepsia 1992 33 04 S15 S25 10.1111/j.1528-1157.1992.tb06223.x 1425490

[18] LaRoche S M Helmers S L Epilepsy in the elderly Neurologist 2003 9 05 241 249 10.1097/01.nrl.0000087719.64343.be 12971835

[19] Ahmad B Wark J D Petty S J Changes in balance function with chronic antiepileptic drug therapy: A twin and sibling study Epilepsia 2015 56 11 1714 1722 10.1111/epi.13136 26513212

[20] Petty S J Hill K D Haber N E Balance impairment in chronic antiepileptic drug users: a twin and sibling study Epilepsia 2010 51 02 280 288 10.1111/j.1528-1167.2009.02254.x 19674052

[21] Ahmad B S Hill K D O'Brien T J Gorelik A Habib N Wark J D Falls and fractures in patients chronically treated with antiepileptic drugs Neurology 2012 79 02 145 151 10.1212/WNL.0b013e31825f0466 22700806

[22] Rowan A J Ramsay R E Collins J F VA Cooperative Study 428 Group New onset geriatric epilepsy: a randomized study of gabapentin, lamotrigine, and carbamazepine Neurology 2005 64 11 1868 1873 10.1212/01.WNL.0000167384.68207.3E 15955935

[23] Gilliam F Carter J Vahle V Tolerability of antiseizure medications: implications for health outcomes Neurology 2004 63(10, suppl 4):S9 S12 10.1212/wnl.63.10_suppl_4.s9 15557551

[24] Drury I Beydoun A Interictal epileptiform activity in elderly patients with epilepsy Electroencephalogr Clin Neurophysiol 1998 106 04 369 373 10.1016/s0013-4694(97)00158-2 9741765

[25] Pohlmann-Eden B Beghi E Camfield C Camfield P The first seizure and its management in adults and children BMJ 2006 332 7537 339 342 10.1136/bmj.332.7537.339 PMC1363913 16470055

[26] Sen A Jette N Husain M Sander J W Epilepsy in older people Lancet 2020 395(10225):735 748 10.1016/S0140-6736(19)33064-8 32113502

[27] Chouksey A Pandey S Functional Movement Disorders in Elderly Tremor Other Hyperkinet Mov (N Y) 2019 9 10.7916/tohm.v0.691 PMC6691911 31413900

[28] Geroin C Petracca M Di Tella S Elderly Onset of Functional Motor Disorders: Clinical Correlates from the Italian Registry Mov Disord Clin Pract 2024 11 01 38 44 10.1002/mdc3.13916 PMC10828615 38291844

